# Solution Growth of Two-Dimensional Bi_2_Se_3_ Nanosheets for Two-Color All-Optical Switching

**DOI:** 10.3390/ma10111332

**Published:** 2017-11-21

**Authors:** Xinghua Wu, Chao Tan, Qingkai Wang, Yanyan Guo, Dianyuan Wang, Yongqian Wang, Dawei Meng

**Affiliations:** 1 Faculty of Materials Science and Chemistry, China University of Geosciences, Wuhan 430074, China; 51205879@163.com (X.W.); yyguo_1980@163.com (Y.G.); cugwyq@126.com (Y.W.); 2 College of Science, Key Laboratory for Microstructural Functional Materials of Jiangxi Province, Jiujiang University, Jiujiang 332005, China; 79792239@163.com (Q.W.); 1064532@163.com (D.W.); 3 School of Information and Electrical Engineering, Hunan University of Science and Technology, Xiangtan 411201, China; chaotanhnu@163.com

**Keywords:** nanoparticles, optical materials and properties, nanocrystalline, optical switching

## Abstract

Two-dimensional Bi_2_Se_3_ nanosheets with hexagonal shape are synthesized by a solution synthetic route. The Bi_2_Se_3_ nanosheets are 120 nm in edge width and 7 nm in thickness. The size of the Bi_2_Se_3_ nanosheets can be controlled by choosing different kinds of reducing agents including hydroxylamine and ethylenediamine. Subsequently, we demonstrate a configuration of two-color all-optical switching based on plasma channels effect using the as-synthesized Bi_2_Se_3_ nanosheets as an optical media. The signal light can be modulated as two states including dot and ring shape by changing the intensity of control light. The modulated signal light exhibits excellent spatial propagation properties. As a type of interesting optical material, ultrathin two-dimensional Bi_2_Se_3_ nanosheets might provide an effective option for photoelectric applications.

## 1. Introduction

Two-dimensional (2D) materials including grapheme [1], transition metal dichalcogenides [2], topological insulators [3] and black phosphorus [4,5] have large lateral size, but small vertical thickness. Due to unique optical, electronic, physical and chemical properties, 2D materials have attracted much attention and have been widely applied in transistors, sensors, batteries, supercapacitors and solar cells [6]. As a 2D material, Bi_2_Se_3_ possesses excellent thermoelectric properties and novel electronic band structure, which are heavily dependent on its morphology and size. Considerable efforts have been made to synthesize well-defined Bi_2_Se_3_ nanostructures via physical vapor deposition [7], molecular beam epitaxy [8], mechanical exfoliation [9] and liquid phase exfoliation [10,11,12,13,14,15]. Solution-based synthesis is an excellent alternative for the preparation of high quality ultrathin 2D Bi_2_Se_3_ nanosheets [16,17,18] and has the advantages of simplicity, low reaction temperature, high yield and large amount [19,20,21,22,23]. The morphology and size of 2D Bi_2_Se_3_ nanosheets can be effectively modulated by changing reaction conditions including surfactants, pH value, reaction temperature and time [12,14,19,24,25]. The reducing agent is necessary and critical for the synthesis of 2D Bi_2_Se_3_ nanosheets. However, searching for an effective reducing agent and establishing the relationship between the reducing agent and the size of nanoparticles are serious challenges.

All-optical switching plays an important role in the all-optical signal processing and optical communication. The performances of all-optical switching heavily depend on optical materials and device configurations [26]. Compared to the traditional nonlinear optical materials, two-dimensional materials have great advantages owing to their strong light-matter interactions, broadband and ultrafast optical responses, large third-order optical nonlinearity [17,27]. Based on spatial self-phase modulation (SSPM) effect, all-optical switching in MoS_2_ and Bi_2_Se_3_ dispersion solution have been realized [26,28,29,30,31,32]. However, self-diffraction ring formation process is slow, and the SSPM pattern is unstable, hindering the application in ultrahigh-speed optical devices. In order to enhance the performance of the device, the search for another effective and reliable approach to realized all-optical switching based on 2D materials is always highly encouraged.

In this paper, we demonstrate the all-optical switching in 2D Bi_2_Se_3_ dispersion solution based on plasma channels effect induced by femtosecond lase. The ultra-thin 2D Bi_2_Se_3_ nanosheets have been successfully synthesized by solution method. The size of the Bi_2_Se_3_ nanosheets can be controlled by choosing different kinds of reducing agents. Using ethylenediamine instead of hydroxylamine as a reducing agent, the lateral size of the nanosheets increases from 100 to 500 nm. Subsequently, the as-synthesized Bi_2_Se_3_ nanosheets were used as optical media for two-color all-optical switching based on plasma channel effect induced by femtosecond lase. Meanwhile, the propagation properties of modulated signal beam with ring-shape in free space are investigated.

## 2. Material Preparation and Experimental Setup

### 2.1. Synthesis of Ultrathin Bi_2_Se_3_ Nanosheets

Bismuth triacetate (Bi(CH_3_CO_2_)_3_, ≥99.99%), Sodium selenite (Na_2_SeO_3_, ≥99%), Hydroxylamine solution (NH_2_OH, 50% in H_2_O) were purchased from Sigma-Aldrich (Shanghai, China). Ethylene glycol (EG), Poly(vinyl pyrrolidone) (PVP, *M*_W_ ≈ 40,000), Ethylenediamine (≥99.5%), Acetic acid glacial (analytical reagent) were purchased from Aladdin (Shanghai, China). Ethanol (analytical reagent) and Acetone (analytical reagent) were purchased from Xilong Chemical Reagent Co. (Shanghai, China). All the chemicals were used as received without further purification.

In a typical synthesis, 0.3 mmol Bi(CH_3_CO_2_)_3_ and 0.4 g PVP were dissolved in 10 mL ethylene glycol in a 25 mL round-bottom flask. Subsequently, 0.45 mmol Na_2_SeO_3_ and 1 mL acetic acid glacial were added into the above solution which was kept stirring evenly until Na_2_SeO_3_ was fully dissolved and a clear solution was obtained. After that, the solution was heated to 170 °C, then a mixture of 1 mL hydroxylamine solution and 1 mL ethylene glycol were rapidly injected, and the solution immediately turned black indicating the formation of Bi_2_Se_3_ nanosheets. The reaction was sustained for 15 min at the above temperature of 170 °C, and then the heating mantle was removed to cool the mixture naturally. After addition of 20 mL acetone, the product was centrifuged at 80,000 rpm for 5 min. The supernatant was discarded and the precipitation was collected. The washing steps were repeated with acetone for two times, and the final product was dispersed in ethanol.

### 2.2. Material Characterizations

Field emission scanning electron microscopy (FESEM, Hitachi S4800, Hitachi Ltd., Tokyo, Japan) was employed to study the morphology of the products. The morphology and microstructure of the as-prepared Bi_2_Se_3_ samples were characterized by high-resolution transmission electron microscopy (HRTEM, FEI Tecnai G2 F20, FEI, Beaverton, OR, USA). The topography and thickness of the as-prepared Bi_2_Se_3_ samples were determined by atomic force microscope (AFM, Bruker, Multimode 8, Camarillo, CA, USA). The crystal structure of the as-prepared Bi_2_Se_3_ samples were investigated by X-ray diffraction using Cu Kα radiation (λ = 1.541 Å) (XRD, D8 ADVANCE, Bruker, Camarillo, CA, USA). Raman spectra of the as-prepared Bi_2_Se_3_ samples were recorded using confocal Raman spectrometer with λ = 532 nm at room temperature (WITec Alpha 300 R, Ulm, Baden-Württemberg, Germany).

### 2.3. Experimental Setup for Two-Color All-Optical Switching

**Figure 1:**
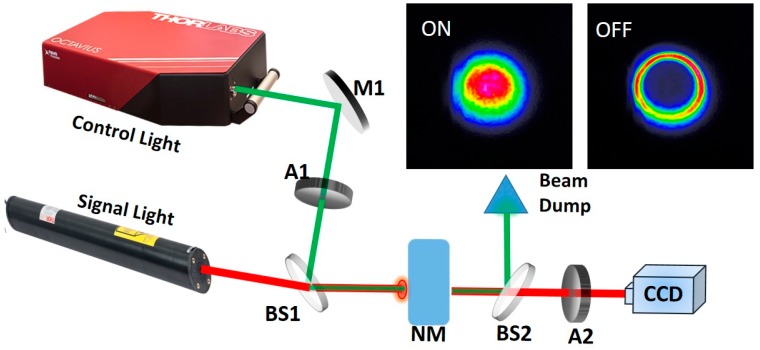
Schematic diagram of proposed two-color all-optical switching configuration. M1, silver-coated plane mirror; A1 and A2, attenuators; BS1 and BS2, beam splitters; NM, nonlinear material (Bi_2_Se_3_ dispersion solution).

Figure 1 has shown the schematic diagram of proposed two-color all-optical switching configuration. In this system, we use continuous wave He-Ne laser with a central wavelength of 632 nm as signal light source. The average output power of signal laser is about 4 mW. A Ti:sapphire amplified laser is used as control light source, which have 126 fs pump pulses, 800 nm central wavelength and 1 KHz repetition rate. The average output power of control light is about 6 mW and can be adjusted by attenuator (A1) from 0 to 6 mW. Profiles of signal and control light are nearly Gaussian, with full width at half maximum (FWHM) values of 1.2 and 0.5 mm, respectively. The Bi_2_Se_3_ nanosheets as nonlinear material filled in a quartz cuvette with 2 cm path length is used as an optical medium. The concentration of Bi_2_Se_3_ dispersion solutions in isopropanol (IPA) is 30, 15, 7.5 and 0 μg/mL. At first, control light passes through an attenuator (A1) to adjust the power. Beam splitters (BS1 and BS2) are dichroic mirrors, they are coated to have high reflectivity at 800 nm and high transmission at 632 nm. Signal and control light converge at BS1, and then collinearly pass through Bi_2_Se_3_ dispersion solution. After passing through BS2, the signal light is separated from control light and get into CCD camera, while the control light is reflected by BS2 and get into the beam dump. The attenuator A2 is used to protect CCD camera from saturation and damage caused by high-powered lasers. Signal light images were captured by a Coherent Laser Cam-HR^TM^ Beamview system (Santa Clara, CA, USA) with 1280 × 1024 pixels and pixel size of 6.7 µm. 

## 3. Results and Discussion

**Figure 2:**
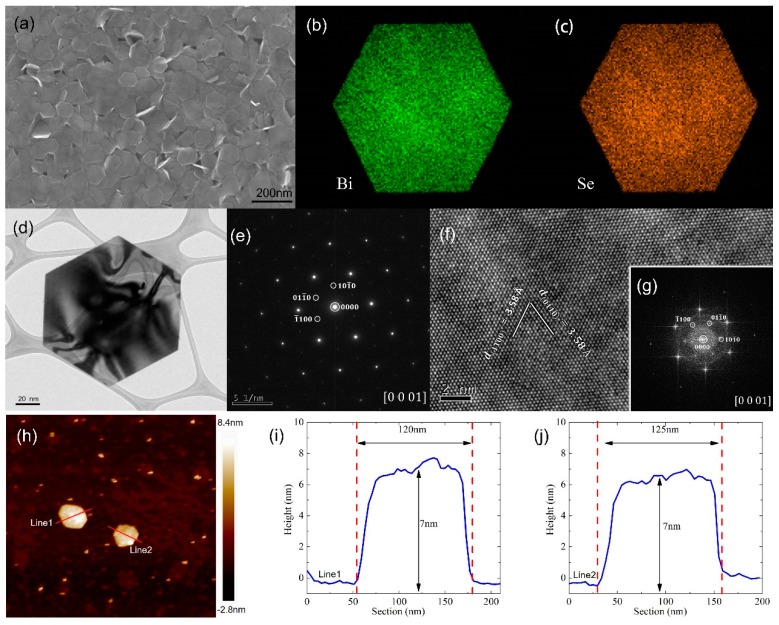
(**a**) Field emission scanning electron microscopy (FESEM) images of Bi_2_Se_3_ nanosheets; (**b**,**c**) EDS elemental mapping images showing the distribution of Bi and Se; (**d**) transmission electron microscopy (TEM) image of Bi_2_Se_3_ nanosheets; (**e**) the corresponding selected area electron diffraction (SAED) pattern; (**f**) the high-resolution transmission electron microscopy (HRTEM) image; (**g**) the Fast Fourier Transform (FFT) pattern; (**h**) atomic force microscope (AFM) image of the Bi_2_Se_3_ nanosheet; (**i**,**j**) AFM height profile corresponding to the line1 and line2 in (**h**).

The morphology of the products was investigated by field emission scanning electron microscopy (FESEM) and transmission electron microscopy (TEM) as shown in Figure 2a–j. The FESEM image (Figure 2a) reveals that the as-synthesized Bi_2_Se_3_ nanosheet through solution method has a very high yield. The nanosheets have regular shape and size, and are predominantly hexagonal morphology. Figure 2d is a typical TEM image of a single Bi_2_Se_3_ nanosheet, which further demonstrates that the as-synthesized nanosheet has perfect hexagonal morphology, and the lateral width of the nanosheet is about 100 nm. In order to make a better analysis to the composition of the nanosheets, Figure 2b,c show energy dispersive X-ray spectroscopy (EDS) elemental mapping images of the samples. Both element Bi and Se have a uniform distribution. The selected area electron diffraction (SAED) pattern shown in Figure 2e can be indexed to a six-fold symmetry [0001] zone axis of the rhombohedral Bi_2_Se_3_. The diffraction spots correspond to the $\left(\bar{1}100\right)$, $\left(10\bar{1}0\right)$ and $\left(01\bar{1}0\right)$ facets of Bi_2_Se_3_ nanosheets, respectively [22,23,24,33,34]. The high-resolution TEM (HRTEM) image of the Bi_2_Se_3_ nanosheets in Figure 2f exhibits high-resolution lattice fringes. The lattice fringes individually correspond to the $\left(\bar{1}100\right)$ facet and $\left(01\bar{1}0\right)$ facet of Bi_2_Se_3_. The Fast Fourier Transform (FFT) electron diffraction pattern of the Bi_2_Se_3_ nanosheets is shown in the inset of Figure 2g. The FFT pattern indicates the $\left(\bar{1}100\right)$, $\left(10\bar{1}0\right)$ and $\left(01\bar{1}0\right)$ facets of Bi_2_Se_3_ nanosheets. The FFT pattern are of six-fold symmetry and can be identified as the projection of the hexagonal Bi_2_Se_3_ reciprocal lattice in [0001] direction. In order to determine the thickness and width of the as-synthesized Bi_2_Se_3_ nanosheets, the atomic force microscopy (AFM) images were carried out. The hexagonal morphology of Bi_2_Se_3_ nanosheets is shown in Figure 2h, and the height profile is shown in Figure 2i,j, which respectively correspond to line1 and line2 in Figure 2h. It is clear that the ultrathin Bi_2_Se_3_ nanaosheet has a thickness of about 7 nm and a uniform width of about 120 nm [13].

**Figure 3:**
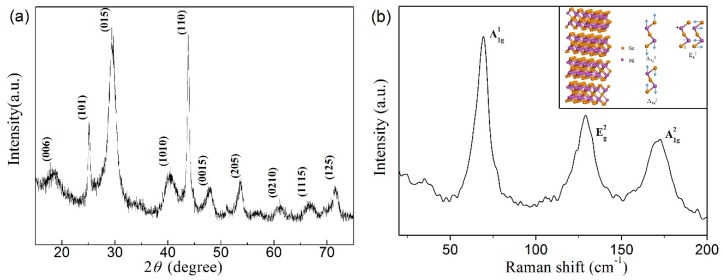
(**a**) X-ray diffraction (XRD) pattern of the Bi_2_Se_3_ nanosheets; (**b**) Raman spectrum of Bi_2_Se_3_ nanosheets. The inset is crystal structure and schematic diagram of the lattice vibrations.

The X-ray diffraction (XRD) pattern of the as-synthesized Bi_2_Se_3_ nanosheets is shown in Figure 3a. All the diffraction peaks can be indexed to rhombohedral Bi_2_Se_3_ structure (space group: $R\bar{3}m$), which are highly consistent with the literature values (JCPDS No. 33-0214) [13,15,35,36]. No peaks of Se or other alloy compounds were detected. Bi_2_Se_3_ is a layered material with a crystal structure of quintuple layers (QL) in which atoms are covalently bonded and stacked in a sequence of Se−Bi−Se−Bi−Se [37]. The QLs are bonded together by weak van der Waals interactions so that ultrathin 2D Bi_2_Se_3_ can be obtained by solution growth route [13,38]. Figure 3b shows the Raman spectrum of the as-synthesized Bi_2_Se_3_ nanosheets [39,40]. The spectrum contains three main peaks (at 70, 128 and 172 cm^−1^) which correspond to A^1^_1g_, E^2^_g_ and A^2^_1g_ modes, respectively [33]. E^2^_g_ mode is in-plane vibrational mode, but A^1^_1g_ and A^2^_1g_ modes are out-of-plane vibrational modes. Out-of-plane vibrational modes are sensitive to the thickness and the intensity increases with reduced thickness [41,42]. The intensity of the A^1^_1g_ mode in the ultrathin 2D Bi_2_Se_3_ nanosheets is relatively strong due to ultrathin thickness of 2D nanosheets [43].

**Figure 4:**
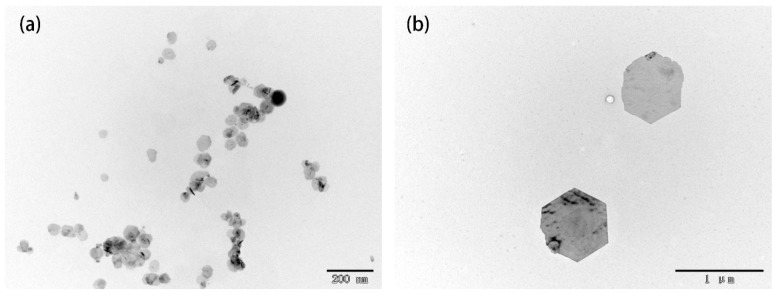
TEM images of Bi_2_Se_3_ nanosheets obtained from the reactions with different reducing agents: (**a**) hydroxylamine; (**b**) ethylenediamine.

The size of Bi_2_Se_3_ nanosheet is controllable by choosing different reducing agents including hydroxylamine and ethylenediamine. During the preparation of Bi_2_Se_3_ nanosheet, when the hydroxylamine is rapidly injected into the precursor solution, the mixture solution immediately becomes black indicating that the Bi_2_Se_3_ nanosheet has been produced and the reaction process is very rapid. With all other reaction conditions unchanged, when the ethylenediamine instead of hydroxylamine is rapidly injected into the precursor solution, the mixture solution first becomes dark brown, then slowly becomes black, incicating that the reaction process is relatively slow. As shown in Figure 4, the width of the Bi_2_Se_3_ nanosheets is about 100 nm for hydroxylamine, and the width is about 700 nm for ethylenediamine.

The reducibility of hydroxylamine is stronger than that of ethylenediamine. When the hydroxylamine is injected into the precursor mixture solution, SeO_3_^−^ is reduced to Se^2−^ which combine with Bi^3+^ to form a large number of crystal nuclei rapidly. The number of crystal nuclei is so large that the growth of the crystal nuclei lacks sufficient power, resulting in the formation of smaller Bi_2_Se_3_ nanosheets [23]. On the contrary, when the ethylenediamine is used, the nucleation rate is slower and the number of crystal nuclei is smaller so that the crystal nuclei fully grow and larger nanosheets are obtained.

**Figure 5:**
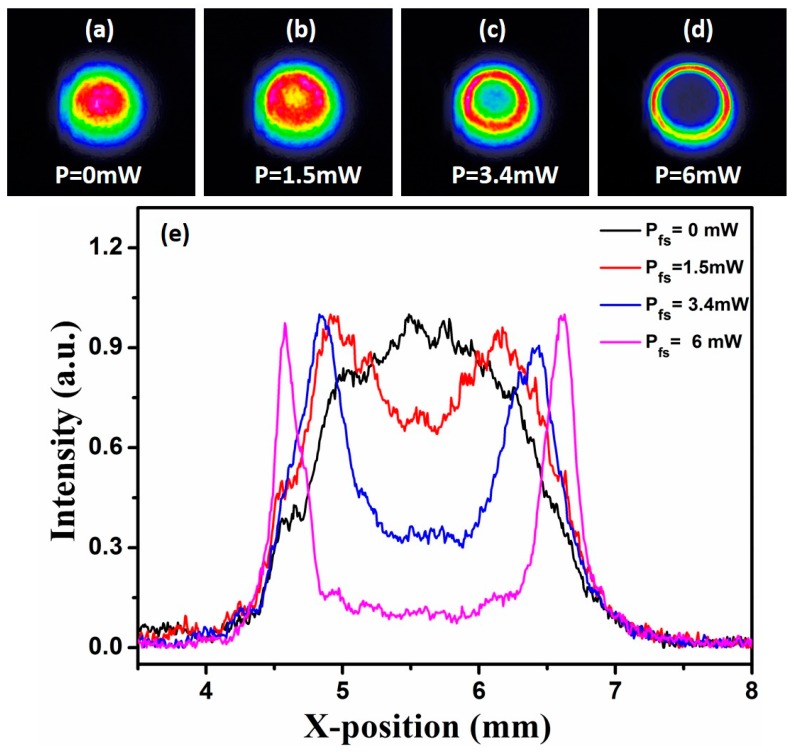
(**a**–**d**) Intensity profiles of signal beams under different P_fs_; (**e**) Radial intensity distribution of signal beams under different P_fs_.

Figure 5a–d shows the beam profiles of the signal light in the Bi_2_Se_3_ dispersion solution at the concentration of 30 µg/mL under different power of control light beams (P_fs_ = 0, 1.5, 3.4, and 6 mW, respectively). In order to further clarify the change of signal light intensity, Figure 5e gives the profile of light intensity distribution through the center of signal light. When the intensities of the control light is fixed at 0 mW, the signal light keeps its initial spatial intensity distribution as Gauss laser spot, and the optical switching is ON. As P_fs_ increases continuously, the light beam collapses toward the center. The intensity of the light spot center decreases gradually, while the intensity of light spot edge increases gradually at the same time. When the control light increases to 6 mW, a dark spot arises in the center region of the signal light, and the optical switching is OFF. The signal light is modulated into a ring shape which remains stable. It can be attributed to laser plasma effect induced by the nonlinearly optical property of the Bi_2_Se_3_ nanosheets. When the control light passes through the Bi_2_Se_3_ dispersion solution, if the power of control light reaches the ionization threshold of the Bi_2_Se_3_ dispersion solution, the plasma channel will be produced. Since the intensity distribution of the control light is Gaussian, the gradient of the electron density and the refractive index distribution in the plasma channel is also approximately Gaussian. The electron density of the plasma in the center of the spot is very high, and the electron density at the edge is low. When the signal light beam passes through the plasma channel, the plasma channel will play the role of a graded-index diverging lens. The signal light beam focuses on the periphery of plasma channel and the intensity of signal light beam is zero in the center.

**Figure 6:**
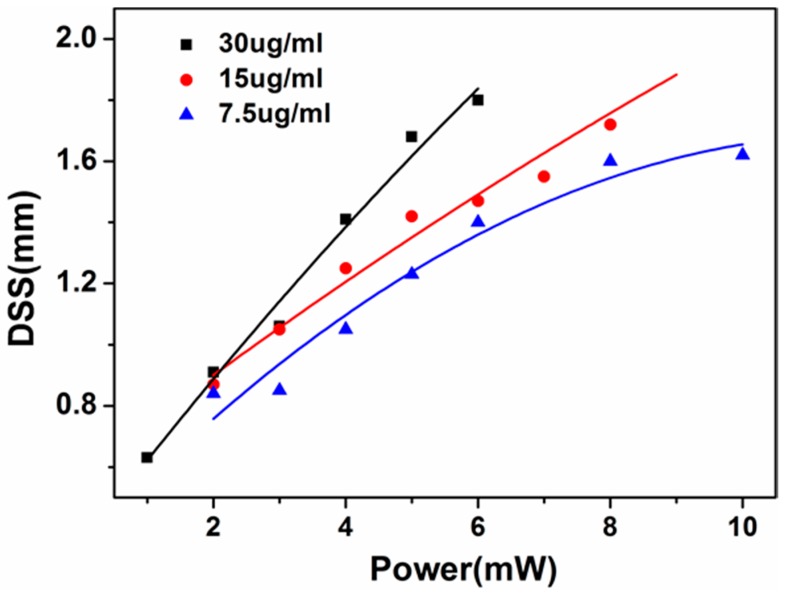
The relationship between the dark spot size (DSS) of signal beams and P_fs_ in three Bi_2_Se_3_ dispersion solutions (30, 15, and 7.5 µg/mL in IPA).

Figure 6 shows the relationship between the dark spot size of signal beams (DSS) and power of control light beams (P_fs_) in three Bi_2_Se_3_ dispersion solutions (30, 15, and 7.5 µg/mL). DSS is defined as the full width at half maximum of the dark spot in the center of signal light beam. In the same concentration of Bi_2_Se_3_, with the increase of power of control light, DSS gradually increase. Meanwhile, as the concentration of Bi_2_Se_3_ increases, to generate the same DSS, the required control light power will decrease. For example, in order to obtain DSS = 1.4 mm, the power of control light required for the Bi_2_Se_3_ dispersion solution with concentration of 30, 15, and 7.5 µg/mL is 4 mW, 5 mW and 6 mW respectively. It can be attributed to that when the concentration of Bi_2_Se_3_ increases, the nonlinear effect is enhanced and the control light power required for the generation of same plasma channels is lower. That is to say, it is easier to obtain more obvious switching effect in high concentration dispersion solution.

**Figure 7:**
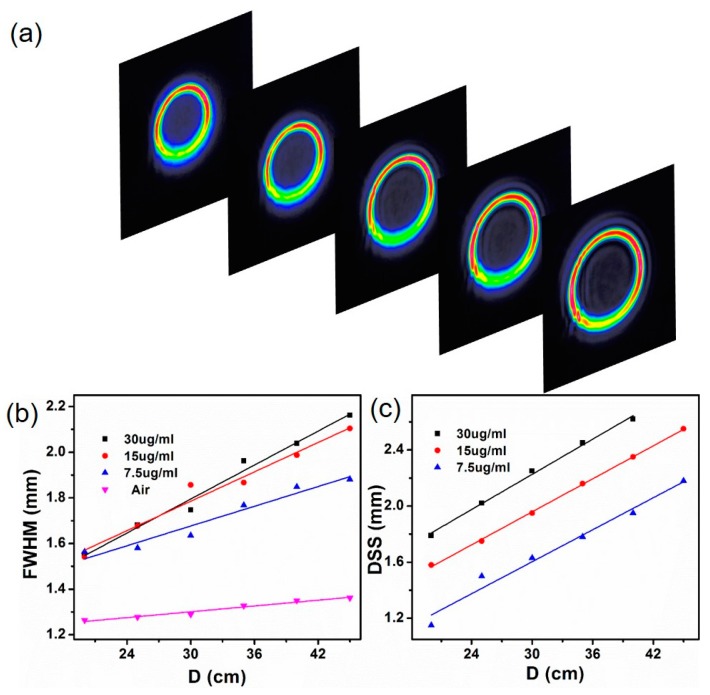
(**a**) Intensity profiles of signal beams propagating in free-space when the switching is OFF; (**b**) Beam widths of signal beams after passing through different mediums at different propagation distances when the switching is ON; (**c**) Relationships between DSS and D when *P*_fs_ is 6 mW and the switching is OFF. The Bi_2_Se_3_ is dispersed in isopropanol (IPA).

To verify the stability of signal beam when the switching is OFF, we study the propagation properties of the signal beam in free space. The signal beam is modulated by the control light beam in the Bi_2_Se_3_ disperse solution to form dark spots. The distance between the Bi_2_Se_3_ dispersion solution and the CCD camera is set to distance D. When the CCD camera moves along the central axis of the signal beam, the distance D changes and a series of spatial intensity distribution patterns are obtained. Figure 7a shows the intensity distribution patterns of signal light beam generated in the Bi_2_Se_3_ dispersion solution (30 µg/mL) at different propagation distance (D) when P_fs_ is 6 mW. With the increase of the distance D, the signal beam keeps initial spatial intensity distribution and exhibits favorable stability in free space.

In order to investigate the propagation of light beam in free space, we further investigated the FWHM of initial signal beam and DSS of modulated signal beam changing with distance D. When the intensities of the control light is 0 mW, the signal light keeps Gaussian intensity distribution, and the optical switching is ON, as shown in Figure 7b. The FWHM of initial signal light beam increases with the increase of distance D. The FWHM of the initial signal beams passing through the Bi_2_Se_3_ dispersion solution are larger than that through the water. The FWHM of the initial signal beam increases gradually with the increase of the concentration of Bi_2_Se_3_ dispersion solution.

Figure 7c displays the relationships between the dark spot size (DSS) of the modulated signal light beam and the propagation distance (D) at different solution concentrations when P_fs_ is 6 mW and the optical switching is OFF. The DSS of the modulated signal beam increases gradually with the increase of distance D. Meanwhile, the DSS of the initial signal beam increases gradually with the increase of the concentration of Bi_2_Se_3_ dispersion solution. It is more easily to achieve control of signal light beam at high concentrations of Bi_2_Se_3_ dispersion solution.

## 4. Conclusions

In summary, two-color all-optical switching has successfully been realized based on two-dimensional Bi_2_Se_3_ nanosheets as an optical media. Two-dimensional Bi_2_Se_3_ nanosheets with highly uniform hexagonal morphology have been successfully synthesized. Then, the as-synthesized Bi_2_Se_3_ nanosheets were dispersed into solution and innovatively used as an optical media for the realization of two-color all-optical switching. It is envisaged that two-dimensional Bi_2_Se_3_ nanomaterials may be utilized as an excellent optical media for all-optical processing toward practical applications, leading to the development of new photoelectric devices.
